# Method of Preserving the Brain

**Published:** 1880-11

**Authors:** 


### METHOD OF PRESERVING THE BRAIN.

Prof. Carlo Giacomini, of Turin, has lately described a process for the preservation of the brain and nerves which promises to be of great utility to the anatomist and pathologist. The description of the process was communicated to the Royal Academy of Medicine, of Turin, on the 7th of June, 1879, and as I lately had an opportunity of seeing the results of the method employed, and was much impressed with its success, I think some account of it may be interesting to the readers of this journal.

The primary induration of the brain may be effected by one or other of several reagents, such as chloride of zinc, chromate of potash, or chromic acid solutions, diluted nitric acid or alcohol; but of all these the saturated solution of chloride of zinc appears for several reasons to be the best.

The newly extracted brain is placed in a saturated solution of the chloride of zinc, with the membrane still adhering to it; and after an immersion for about forty-eight hours the membranes and blood-vessels may with ease be removed, care being taken to preserve the surface as entire as possible. The brain at first naturally floats in the solutions, so that it must be turned in it from time to time till the whole is acted upon, and from the increase in specific gravity it at last sinks in the solution. This generally occurs after two or three day's further immersion, and then the brain is to be transferred from the solution to alcohol of commerce. By this part of the process the brain loses about a twentieth or twenty-fifth part of its weight.

Should there be reason to believe that the brain has suffered from the body having been kept too long after death, or if it is wished to examine it *in situ*, the induration may be effected by the injection of 600 grammes of the saturated chloride of zinc solution in the internal carotid arteries.

The brain when transferred to the alcohol sinks in the fluid, and precautions must be taken, by frequently changing its position, to prevent any alteration of form by pressure against the sides or bottom of the vessels in which it is laid. After remaining ten or twelve days in the alcohol, the brain is ready to be transferred to glycerine.

For this purpose white or colorless fenicated glycerine (in the proportion of one to 100) is found to answer best. The brain must remain in the glycerine for a number of days till it is thoroughly impregnated with it, when it will be found to have regained from five to six and one half ounces in weight. It is then removed from the glycerine, and placed in any convenient situation, to allow of some desiccation and exudation of superfluous fluid, and it may either be preserved in this state, or, what is better, it may be covered with one or more layers of a varnish of caoutchouc or marine glue, which, while it does not materially alter its appearance or consistance, protects the surface from the adhesion of dust and from mechanical injury, and enables the preparation to be handled perfectly freely without risk of breaking the surface. The final varnishing is not, however, necessary if the preparations are otherwise fully protected.

Preparations made after the manner now described may be kept for years in close glass or other cases without change; and such is the hygroscopic property of the glycerine that they may even be left exposed for a considerable time in ordinary states of the atmosphere without undergoing any marked alteration in weight or appearance. The natural form and color of the brain are in great measure maintained, and even the distinction of the gray and white substance, and the fibrous character of the latter are preserved in such a manner that thin slices can be made, and dissections of the preserved brains, much the same way as in a fresh brain, or in one which has been successfully preserved in alcohol.

It is true the microscopic character of minute structures are not perfectly preserved; but this could not have been expected from any other methods than those adopted by histologists in their improved modern processes. But for the conservation of series of human brains and those of animals for purposes of exhibition and demonstration, and for the study of the external form and larger structure, they are admirably adapted and likely to prove most valuable, for they remain to some extent soft and pliable, can be handled with freedom, and may be dissected or examined in every way that may be desired.

It is obvious that series of brains so preserved are remarkably well adapted for the study of the comparative anatomy and homology of the sulci and convolutions both in man and animals, and that they are equally well suited for serving as records of the local effects of pathological changes or of physiological experiments.

